# Prospective Clinical Trials to Advance the Study of Immune Checkpoint Inhibitor Toxicity

**DOI:** 10.3390/curroncol30070502

**Published:** 2023-07-20

**Authors:** Christopher Cluxton, Jarushka Naidoo

**Affiliations:** 1 Beaumont Hospital, RCSI Cancer Centre, D09 V2N0 Dublin, Ireland; cluxtoc@tcd.ie; 2 Department of Medicine, RCSI University of Medicine and Health Sciences, D02 YN77 Dublin, Ireland; 3 Sidney Kimmel Comprehensive Cancer Center at John Hopkins University, Baltimore, MD 21218, USA

**Keywords:** cancer immunotherapy, tumour immunology, clinical trials, adverse effects

## Abstract

Immune checkpoint inhibitors (ICIs) are a class of drug that produces durable and sustained anti-tumour responses in a wide variety of malignancies. The exponential rise in their use has been mirrored by a rise in immune-related adverse events (IrAEs). Knowledge of such toxicities, as well as effective management algorithms for these toxicities, is essential to optimize clinical efficacy and safety. Currently, the guidelines for management of the IrAEs are based largely on retrospective studies and case series. In this article, we review the current landscape of clinical trials investigating the management of IrAEs with an aim to develop standardised, randomised controlled trial-based management algorithms for ICI-related toxicities.

## 1. Introduction

Cancer immunotherapy is based on the principle that a patient’s own immune system can be harnessed to reject a malignant tumour. The concept of immunoediting, which holds that many early cancers are eliminated by immune surveillance, is supported by experiments with immunodeficient mice and epidemiologic studies of immunocompromised individuals [1]. Cancer cells must become less immunogenic or disable immunologic components to survive and spread throughout the body. There are numerous cancer immunotherapy techniques that are currently being used in the clinic or under development, such as cytokines, cellular therapies, viral vectors for gene transfer, and antibody-based therapies [2]. Collectively, these therapeutic modalities represent a paradigm shift in cancer treatment by targeting key pathways and cell types within the host immune response, rather than the cancer cell, and have been successful in improving clinical outcomes for patients with both solid tumour and haematologic malignancies [3,4,5,6].

The antigen-specific T-cell receptor and accessory receptors are required for the transmission of signals that activate T-cells [7]. These accessory receptors serve to enhance or inhibit TCR-mediated signals. The accessory receptors CTLA-4 and PD-1, which are expressed on activated T-cells, function as negative regulators to suppress T-cell responses [8]. In general, the term “immune checkpoint blockade” refers to a therapeutic strategy that facilitates T-cell immunity by using antagonistic monoclonal antibodies [9].

Immune checkpoint inhibitors modulate the immune system and in doing so can precipitate a unique set of side effects known as immune-related adverse events (IrAEs). These differ from toxicities observed with traditional chemotherapeutic agents owing to their immunologic mechanisms [10,11,12]. IrAEs can affect the skin (for example, vitiligo and autoimmune dermatitis) [12], gastrointestinal (GI) tract (colitis) [13], lungs (pneumonitis) [14,15,16], endocrine organs (thyroiditis, hypo- or hyperthyroidism, primary adrenal insufficiency, diabetes mellitus, or hypophysitis) [17,18], musculoskeletal system (arthritis, myositis) [19], kidneys (nephritis) [20], liver (hepatitis) [21], central or peripheral nervous system (neuropathy, encephalitis) [22], and eyes (uveitis/iritis) [23]; however, any organ system can be involved. IrAEs across organ systems or within an organ system may have different natural histories with respect to time of onset, clinical course, outcome, or response to treatment. More specifically, most IrAEs appear to be acute and can be managed effectively with anti-inflammatory therapies. Other IrAEs may become chronic, with the presence of antibodies, or may have a prolonged inflammatory phase resulting in chronic inflammatory disease [24]. The management of high grade IrAEs often requires guidance from multidisciplinary specialists [25,26,27]. Systemic corticosteroid administration is the main method of treatment for IrAEs; however, there are other strategies that can be used, such as supportive care, additional immunosuppression, and treatment interruption or delay.

## 2. Mechanisms of Immune-Related Toxicity

Immune-related adverse events may be mediated by different mechanisms, including T-cells, autoantibodies, cytokines, HLA-predisposition, and the microbiome [28]. It is known that increasing T-cell activity against antigens that are present in tumour and healthy tissue, increasing levels of existing antibodies, increasing levels of inflammatory cytokines, and enhancing complement-mediated inflammation result in toxicity. Different checkpoint inhibitors also affect tissues in different manners, to different degrees; for example, patients treated with anti-CTLA-4 therapy or anti-PD-1 therapy experience differences in organ-specific toxicities. CTLA-4 therapy is associated more commonly with colitis and hypophysitis, while thyroiditis and pneumonitis are more commonly observed in patients treated with anti-PD-1 therapy [15,29]. IrAEs are common, with toxicity of any grade occurring in up to 90% of patients treated with the anti-CTLA-4 antibody and up to 70% of patients treated with PD-1 or PD-L1 antibodies [30]. There is, however, a wide variety of tumour types with varying immune microenvironments resulting in different patterns of incidence, type, and grade of IrAE. A meta-analysis of patients with cancer, who received therapy with nivolumab, pembrolizumab, atezolizumab, durvalumab, and avelumab revealed an incidence of IrAEs of any grade of 26.8% and high/severe grade of 6.1% globally. This analysis represented more than 12,000 patients in 46 studies. Additionally, the organ-specific toxicities were variable for each agent; for example, skin involvement was the most prevalent site of IrAEs of all grades induced by nivolumab, followed by the gastrointestinal tract, endocrine system, liver, lung, and kidney. Of note, severe grade events induced by nivolumab were observed most commonly in the gastrointestinal tract and liver [30]. The spectrum is also different for combination treatment with multiple ICIs, with chemotherapy, and/or with tyrosine kinase inhibitors. When immune checkpoint blockage is combined, they are both more prevalent and more severe. The reason why IrAEs arise in some people but not in others is not entirely understood. The potential risk factors for IrAEs have been examined in several studies, including autoimmune disease history, age, ethnicity, elevated body mass index, genetic variables, and differences in the microbiologic makeup of the patients’ gut flora [31,32,33].

## 3. Management of IrAEs

Immune-related adverse events result from host immune response directed against normal organs. As a result, the mainstay of treatment in the acute setting is immunosuppression with oral corticosteroids, high-dose steroid therapy, or additional immunosuppressants in more severe or refractory cases [34]. For steroid-refractory cases, early initiation of additional immunosuppressants or plasmapheresis can be considered. This is often under the close guidance of disease-specific subspecialists [34]. Examples of immunomodulatory agents that may be used for IrAE management include infliximab, tumour necrosis factor inhibitors (TNFi’s), mycophenolate mofetil, anti-thymocyte globulin (ATG), calcineurin inhibitors, methotrexate, or intravenous gamma-globulin (IVIG) [34]. The management of immunotherapy toxicities is guided by Common Terminology Criteria for Adverse Events (CTCAE–Version 5.0) grade of severity [35], type, and number of adverse events [25,26,27].

In general, immunotherapy treatment can be continued with close monitoring for grade 1 IrAEs [36]. For grade 2 IrAEs, corticosteroid treatment with 0.5–1 mg/kg prednisone/equivalent is recommended. In such cases, immunotherapy should be withheld until resolution of the toxicity to a severity less than or equal to grade 1. For grade 3 IrAEs, immunotherapy should be discontinued. However, in selected circumstances or in the case of endocrine toxicities not treated with steroids, retreatment with ICIs may be considered when IrAEs improve to grade ≤ 1. In severe grade cases, treatment with high-dose steroids (oral prednisolone 1–2 mg/kg/day or IV equivalent) should be commenced with a slow taper over 4–6 weeks. Longer steroid tapers (6–8 weeks or more) may be required, especially in the case of pneumonitis and hepatitis [14,34,37].

Additionally, there is a fine balance that must be struck between treatment efficacy and toxicity. Several studies report that there is a direct correlation between the development of IrAEs and the response of a tumour to ICIs. Indeed, this is also true across several tumour types [38,39,40]. This is likely due to both being linked to a more robust immune response overall. Conversely, there are studies that report poorer outcomes for patients who develop early IrAEs, including pneumonitis in patients treated with ICIs (nivolumab or pembrolizumab) for lung cancer [41]. It has also been a concern that treating IrAEs with steroids may dampen or counteract the anti-tumour function of ICIs; however, retrospective studies suggest that this is, in fact, not that case [42]. A caveat to this appears to be patients commencing ICI therapy while already taking steroids, who have demonstrably worse outcomes than those not on steroid therapy [43]. This complex and nuanced relationship between treatment and toxicity clearly highlights the importance of high-quality clinical data in the development of guidelines for the effective treatment of cancers with ICIs.

## 4. Clinical Trials in IrAEs

In addition to their use in comparing novel immunotherapy treatments to the current standard of care, clinical trials have been developed and initiated to understand aspects of the safety of ICIs, including the natural history of IrAEs and the safety and efficacy of ICIs in high-risk populations, including those with pre-existing autoimmune disease and solid organ transplant [44,45,46].

Patients with autoimmune disease often experience symptoms of local or systemic inflammation, and if these symptoms are in the same organ, it may constitute a "‘flare’ phenomenon. The use of immunotherapeutic agents in patients with both malignancy and autoimmune disease may result in a greater incidence or severity of IrAEs or may induce exacerbations of the underlying autoimmune disease itself [10,19,24]. As a result, the co-existence of an autoimmune disease is a common exclusion criterion in clinical trials [33]. Resultantly, there is lack of evidence on the safety of immunotherapeutic agents in this setting, and there is no framework for the management of such patients. A phase Ib observational trial is ongoing, exploring the use of nivolumab in patients with autoimmune disorders and advanced, metastatic, or unresectable cancer to assess safety. This includes the incidence of dose-limiting toxicity (DLT) and other toxicities associated with the use of nivolumab in patients with varying severity of common autoimmune disorders (NCT03816345).

Patients with solid organ transplants, such as renal transplants, represent another high-risk group for ICI treatment for cancer [46]. It is well-established that patients with kidney transplants are at a higher risk of developing cancer than the general population, primarily due to long-term immune suppression to prevent immune-mediated transplant rejection [41]. Renal transplantation is generally an exclusion criterion, and case study evidence suggests that 20–50% of patients experience transplant rejection from immunotherapy mediated by T-cell targeting of the graft [47]. A phase I clinical trial (NCT03816332) was developed in 2019 to explore the anti-tumour efficacy and safety of the combination of tacrolimus, nivolumab, and ipilimumab patients with kidney transplants and unresectable or metastatic cancer suitable for ICI therapy. The primary objective of this ongoing trial is to estimate the proportion of patients with kidney transplants and advanced cancers suitable for ICI as a standard therapy who would derive clinical benefit from prednisone, tacrolimus, and nivolumab, without allograft loss. The results of this trial would, therefore, inform clinicians on the safety of nivolumab on graft rejection and provide data on the efficacy of treatment in this high-risk population.

### 4.1. Colitis

Gastrointestinal tract (GI) IrAEs, such as diarrhoea/colitis, can be common; for example, 27–31% of patients treated with ipilimumab monotherapy will experience gastrointestinal tract symptoms of any grade, rising to 45% for those on combination therapy [13,21,48,49]. Life-threatening diarrhoea/colitis (i.e., grade 3–4) can occur in approximately 3% of patients receiving nivolumab monotherapy, 6% of patients receiving ipilimumab monotherapy, and 9% of patients receiving dual therapy with ipilimumab and nivolumab [13,21]. Holding immune checkpoint inhibitor medication, initiating high-dose steroids (1–2 mg/kg/d), and adding infliximab or vedolizumab in cases where symptoms are steroid-refractory are the conventional treatments for severe immune-related diarrhoea/colitis. Although not all patients require treatment beyond steroids to achieve colitis remission, the early inclusion of secondary immunosuppression may lower hospitalizations and increase the likelihood of a successful steroid taper for the immune-related side effects of diarrhoea/colitis. In 2009, Weber et al. published a randomised, double-blind, placebo-controlled phase II study comparing the tolerability and efficacy of ipilimumab with or without budesonide for unresectable stage III or IV melanoma in which they demonstrated that ipilimumab shows activity in advanced melanoma, with encouraging survival and manageable adverse events. They also concluded that budesonide should not be used prophylactically for grade > or =2 diarrhoea associated with ipilimumab therapy, as it was proven to be effective in this study population [50].

There are several ongoing trials aiming to explore more specific methods to treat immune-related diarrhoea/colitis beyond corticosteroids. For example, a phase I/II clinical trial (NCT04407247) is testing the use of either infliximab or vedolizumab in patients who develop immune-related colitis after immunotherapy for either genitourinary cancers or melanoma after failure of corticosteroids (Table 1).

Additionally, in 2021, a phase II interventional, open-label, randomised trial (NCT04797325) to assess the efficacy and treatment duration of vedolizumab for immune-mediated colitis was initiated and enrolled 84 patients (Table 1). The underlying hypotheses are that in the setting of immune-related colitis, vedolizumab induces the remission of colitis, reduces progression from grade 2 to more severe colitis, decreases the need for corticosteroids, is not associated with severe adverse events or increased risk of tumour progression, and allows the reintroduction/continuation of immunotherapy. This prospective trial will inform on the benefit of vedolizumab in the management of immune-related colitis and as a corticosteroid-sparing therapy.

Lastly, tofacitinib, a JAK kinase inhibitor, is an established therapy for ulcerative colitis refractory to corticosteroid based on the OCTAVE studies (NCT01470612; Table 1) [51,52,53,54]. Subsequently, it was reported in a single case to treat effectively immune-related colitis secondary to pembrolizumab treatment that was refractory to corticosteroids, infliximab, and vedolizumab [55]. A single-arm phase II pilot study (NCT04768504) was, thus, developed to evaluate the efficacy and safety of tofacitinib in patients with immune-related colitis from ICI therapy who have failed corticosteroid and at least one biologic therapy. The primary objective of this study is to determine the efficacy of tofacitinib in inducing clinical remission of immune-related colitis, as measured by the proportion of patients who experience diarrhoea resolution to grade ≤ 1 by CTCAE v5.0, without the requirement for additional immunosuppression (e.g., corticosteroids, biologics, or other immunosuppressors targeted for colitis) 8 weeks post-first-dose of tofacitinib. The data from these trials will refine the management of ICI colitis and allow a more tailored, structured, and evidence-based approach.

### 4.2. Pneumonitis

Pneumonitis is defined as inflammation of the lung parenchyma. It is the most common pulmonary toxicity associated with immunotherapy and has variable clinical presentation, severity, and radiological findings [14]. Immunotherapy-related pneumonitis, also called checkpoint inhibitor pneumonitis (CIP), is most common in patients with non-small-cell lung cancer (NSCLC) and renal cell carcinoma (RCC). It is also more frequently observed with PD-1/PD-L1 therapy than with CTLA-4 inhibitors, although the highest rates are observed with dual therapy [56]. The severity of CIP ranges from asymptomatic to respiratory compromise and has a mortality rate of more than 10% of cases [37]. Thus, an essential part of monitoring patients taking ICIs is identifying CIP and starting the appropriate treatment in a timely manner. CIP can, however, be difficult to diagnose, as radiological appearances can mimic disease progression, infection, and chemoradiation-related pneumonitis [57]. Therefore, patients with new respiratory symptoms, a new oxygen requirement, or a reduction in functional status should be assessed with an urgent CT thorax. Acute interstitial pneumonitis/diffuse alveolar damage syndrome is the most acute life-threatening event, and patients commonly experience pneumonitis several months after treatment, which is later than other IrAEs [57].

The management of pneumonitis is similar to other IrAEs in that mild cases are managed by withholding therapy, and more severe cases are managed with corticosteroids. Other forms of immunosuppression may be used in severe or refractory cases, and typical treatments include infliximab, cyclophosphamide, or mycophenolate mofetil [58]. Additionally, for more severe grades of pneumonitis, consultations from infectious disease and pulmonary physicians may be appropriate to rule out infectious causes and to conduct investigations, including pulmonary function testing and bronchoscopy.

The Optimizing Immunosuppression for Steroid-Refractory Anti-PD-1/PD-L1 Pneumonitis trial (NCT04438382) is a phase II, open-label, randomised, interventional trial to optimise immunosuppression for steroid-refractory anti-PD-1/PD-L1 pneumonitis by studying the comparative effects of infliximab and intravenous immunoglobulin (Table 1). This trial examines infliximab and intravenous immunoglobulin treatment of steroid-refractory pneumonitis. It is currently unknown whether infliximab and intravenous immunoglobulin therapy improves pneumonitis. Given the severity and high mortality rates of ICI-related pneumonitis, early intervention with the most effective agent is essential for favourable clinical outcomes. This trial will, therefore, inform the best strategy to manage steroid-refractory ICI pneumonitis, an area in which little is known.

### 4.3. Myocarditis

Traditional cytotoxic chemotherapy and novel cancer therapies may result in a variety of cardiotoxicities, ranging from heart failure to arrhythmias. In contrast to more common IrAEs that have a low associated mortality of 2–5%, myocarditis has a high rate of mortality of 20–50% [59,60,61,62]. ICI-related myocarditis has a reported incidence of 0.04% to 1.14%. Indeed, combination therapy almost doubles the incidence of and mortality from myocarditis [37]. The time to onset of immunotherapy-induced myocarditis is also highly variable, with Mahmood et al. describing a median onset of 34 days, and 81% of cases presenting in the first three months, with reports of presentations as delayed as 450 days [60]. A second study published by Escudier et al. described a time-to-onset range of 2 to 454 days, with a median of 65 days [63]. There was an average of three infusions administered before cardiotoxicity diagnosis [63]. These data, therefore, suggest that most cases will present within the first 2 months, although the diagnosis should still be considered for all patients on immunotherapy with relevant and significant symptoms.

The exact mechanism of ICI-related myocarditis is unclear. Suggested mechanisms include a shared antigen between the tumour and myocardium, T-cell receptor targeting a different but homologous muscle antigen as the tumour antigen, or certain T-cell receptors targeting dissimilar antigens [59]. It is possible that the mechanism may be analogous to the proposed mechanisms of viral-mediated myocarditis, i.e., the tumour and myocardium have similar antigens that are targeted in a mechanism of molecular mimicry [64]. This mechanism would support the finding that that the myocardium displays histological infiltration by T lymphocytes [59]. There have also been reports that PD-L1 is expressed on the myocardium of patients with immune-related myocarditis, supporting this hypothesis [59].

Similar to other IrAEs, myocarditis is managed primarily with corticosteroid, tailored to grade severity [65,66,67,68]. Steroid-refractory or severe immune-related myocarditis has been treated successfully with additional immunosuppressants, including intravenous immunoglobulin, mycophenolate, infliximab, anti–thymocyte globulin, plasmapheresis, alemtuzumab, and abatacept [66,67,69,70,71]. Indeed, the American Heart Foundation (AHA) recommends the use of additional immunosuppressants in such cases. Additionally, a multi-disciplinary team approach to managing higher grade cases of ICI-related myocarditis is advised, to include the early consultation with cardiac specialists.

The AbatacepT foR ImmUne Checkpoint Inhibitor Associated Myocarditis (ATRIUM) trial (NCT05335928) is a phase III investigator-initiated, randomised, double-blind, placebo-controlled study to assess the efficacy and safety of abatacept in immune-related myocarditis (Table 1). The investigator’s objective is to examine whether abatacept reduces major adverse cardiac events (MACE) in cases of immune-related myocarditis. Abatacept functions to suppress T lymphocyte activation by binding CD80 and CD86, blocking its interaction with CD20 [66]. In animal models of myocarditis, abatacept reduced cardiac autoimmunity and increased survival. However, the evidence for abatacept for patients with immune-related myocarditis is lacking. Therefore, the ATRIUM study aims to assess the effect of abatacept concurrently administered with corticosteroid in MACE in patients with recently diagnosed immune-related myocarditis. The authors estimate enrolment of 390 patients, and the trail will continue for 5 years. The results of this trial will provide evidence on the use of abatacept in the management of ICI myocarditis. Additionally, in 2023, Salem et al. published a strategy for the identification of individuals with severe ICI myocarditis by also screening for and managing concomitant respiratory muscle involvement, as well as treatment with CTLA4-fusion protein abatacept and the Janus-kinase inhibitor ruxolitinib. Forty cases with ICI myocarditis were included with concomitant myositis in most patients. In the first 10 patients, following recommended guidelines, myotoxicity-related fatality occurred in 60% of the patients. In the subsequent 30 cases, active ventilation and treatment using ruxolitinib and abatacept reduced mortality to 3.4% [1/30] vs. 60% in the 1st quartile (*p* < 0.0001) [72].

## 5. Discussion

The discovery of immune checkpoint proteins represents a significant breakthrough in the field of cancer immunotherapy. However, not all patients respond favourably to these drugs, and many develop IrAEs. IrAEs range in severity from mild to severe and can often be life-threating, resulting in death. In cases of moderate to severe toxicity, the ICI, which may be providing measurable clinical benefit, is often withheld. The standard treatment of such IrAEs is with corticosteroids, either orally or intravenously, with limited evidence for alternative immunosuppressive agents, including infliximab, cyclophosphamide, mycophenolate, and intravenous immunoglobulin. The evidence for the use of these agents comes primarily from case study series, retrospective studies, and personal experience of the treating physician. The gold standard test to examine the safety and efficacy of these agents is the prospective double-blind, randomised, interventional clinical trial, and a number of these have been initiated over recent years for various agents and various IrAEs.

In this narrative review, we provide a comprehensive overview of prospective trials that aim to improve our understanding of how to manage IrAEs. For IrAEs, numerous interventional trials have been recently initiated, including those for myositis, colitis, and pneumonitis, as previously discussed. Without such prospective trials, the field would continue to rely heavily on case study series and retrospective data and guidelines, which are based primarily on the same.

The role and need for well-designed randomised, interventional clinical trials are well known; however, the field has been slow to develop these for several reasons. The management of IrAEs requires multidisciplinary teams, which are largely not set up. As a result, organ specialists and oncologists may conduct trials independently. Additionally, trials on difficult-to-treat, corticosteroid-refractory IrAEs are more challenging to conduct, resulting in longer accrual periods. This results in clinicians often treating patients in the absence of guidelines that are driven by the highest level of evidence. In progressing the field, it is essential that prospective interventional studies are developed to study the comparative safety and efficacy of agents to treat IrAEs and to update guidelines to ensure that the best practice is standardised for the benefit of all patients receiving ICI treatment. Additionally, where possible, observational studies will complement these and may assist us to better understand the effects of ICIs in at-risk populations.

ICI treatment has completely transformed cancer immunotherapy. The discovery of immune checkpoints, such as CTLA-4 and PD-1, has unquestionably aided the advancement of cancer immunotherapy. They provide a marked, and often sustained, response in patients with metastatic disease. Given the high rates of IrAEs and the current lack of clinical trial data to support their management, it is imperative that more clinical trials are initiated to help significant morbidity and mortality secondary to these agents.

## Figures and Tables

**Table 1:** Clinical trials for the management of IrAEs.

IrAE	Intervention	Cancer Type	Immune Checkpoint Inhibitor	Trial Type	Sample Size	Phase	Trial Reference Code
Colitis	Infliximab or Vedolizumab	Genitourinary cancers or Melanoma	Any FDA-approved	Randomised, open-label, interventional	100	I/II	NCT04407247
	Vedolizumab or Prednisolone	Solid tumours	PD-1, PD-L1, and/or CTLA-4	Randomised, open-label, interventional	82	II	NCT04797325
	Tofacitinib	Solid tumours	Any FDA-approved	Single group, open-label, interventional	10	II	NCT04768504
Pneumonitis	Infliximab or Intravenous immunoglobulin	Any	Anti-PD-1/PD-L1 agent (alone or in combination)	Randomised, open-label, interventional	36	II	NCT04438382
Myocarditis	Abatacept	Any	Any FDA-approved	Randomised, quadruple masked, interventional	390	III	NCT05335928
